# Nephrocytes are part of the spectrum of filtration epithelial diversity

**DOI:** 10.1007/s00441-020-03313-7

**Published:** 2020-11-16

**Authors:** Takayuki Miyaki, Yuto Kawasaki, Akira Matsumoto, Soichiro Kakuta, Tatsuo Sakai, Koichiro Ichimura

**Affiliations:** 1 Department of Anatomy and Life Structure, Juntendo University Graduate School of Medicine, Tokyo, Japan; 2 Department of Biology, Juntendo University School of Medicine, Inzai, Chiba Japan; 3 Laboratory of Morphology and Image Analysis, Center for Biomedical Research Resources, Juntendo University Graduate School of Medicine, Tokyo, Japan

**Keywords:** 3D ultrastructure, Podocytes, Nephrocytes, Decapod, FIB-SEM tomography

## Abstract

The excretory system produces urine by ultrafiltration via a filtration epithelium. Podocytes are widely found as filtration epithelial cells in eucoelomates. In some animal taxa, including insects and crustaceans, nephrocytes serve to separate toxic substances from the body fluid, in addition to podocytes. *Drosophila* nephrocytes have been recently utilized as a model system to study podocyte function and disease. However, functionality and cellular architecture are strikingly different between *Drosophila* nephrocytes and eucoelomate podocytes, and the phylogenetic relationship between these cells remains enigmatic. In this study, using focused-ion beam-scanning electron microscopy (FIB-SEM) tomography, we revealed three-dimensional architecture of decapod nephrocytes with unprecedented accuracy—they filled an enormous gap, which can be called “missing link,” in the evolutionary diversity of podocytes and nephrocytes. Thus, we concluded that nephrocytes are part of the spectrum of filtration epithelial diversity in animal phylogeny.

## Introduction

The excretory system plays an important role in the homeostatic regulation of body fluid in multicellular animals (Andrikou et al. 2019; Evans 2008). This organ system initially generates primary urine by filtration of body fluid through a filtration epithelium. The primary urine is subsequently modified by the secretory and absorptive functions in the modulating tubule and excreted as terminal urine (Supplementary Fig. S1) (Ichimura and Sakai 2017; Ruppert et al. 2003).

Eucoelomates, which have the coelom or coelomic sac laying the mesothelium, develop part of the mesothelium into the filtration epithelium composed by podocytes (Ruppert and Smith 1988). Primary urine excluded into the coelomic lumen via the podocyte-based filtration epithelium enters into the nephridium, i.e., a modulating tubule opening to the coelomic cavity (Supplementary Fig. S1). In vertebrates, the Bowman’s capsule, which contains the podocyte-based filtration epithelium, can be regarded as a micro-coelomic sac newly formed in the mesonephric and metanephric kidneys (Ichimura and Sakai 2018; Ruppert 1994) (Supplementary Fig. S1).

**Fig. 1:**
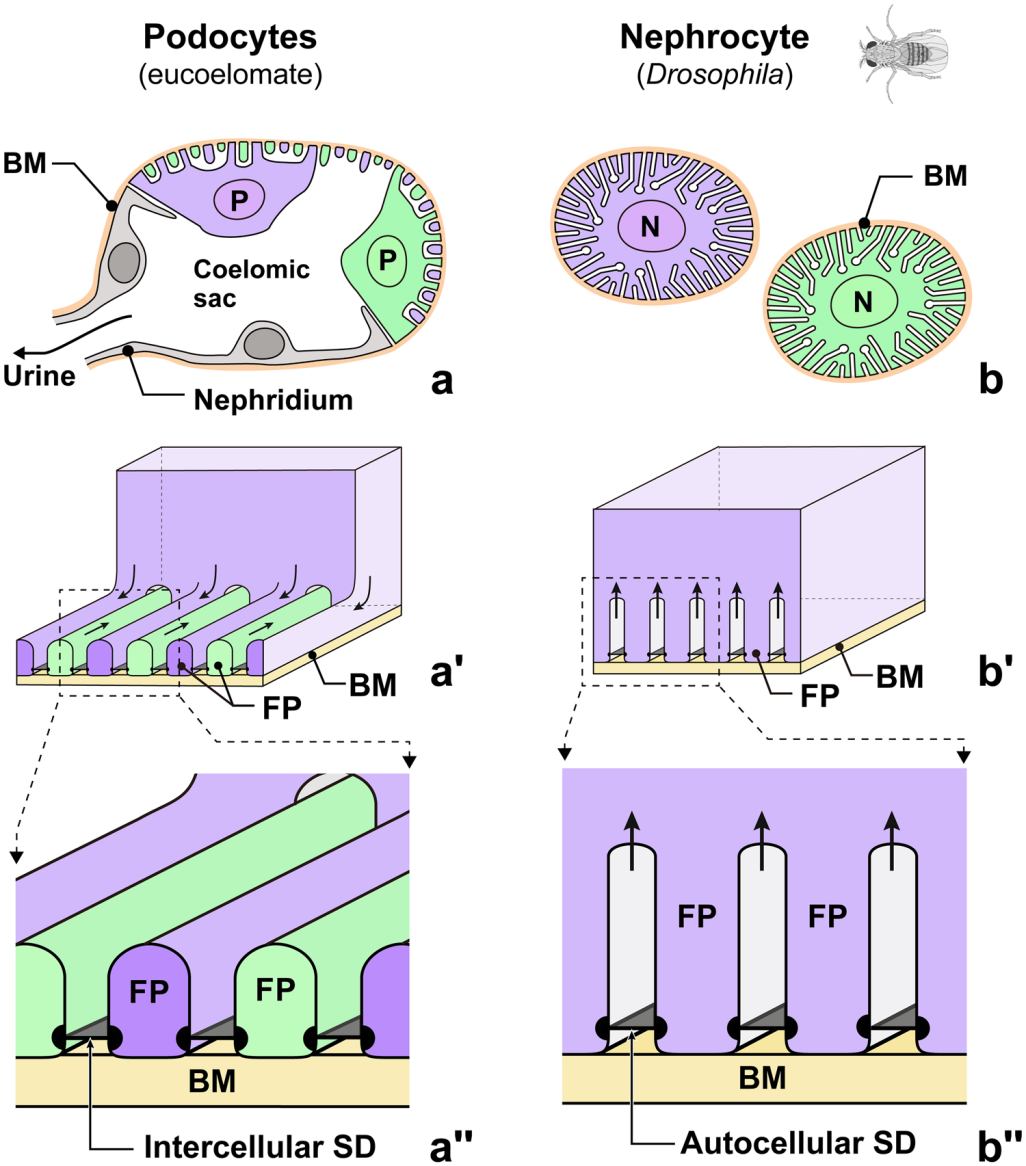
Structural difference between eucoelomate epithelium-forming podocytes and *Drosophila* solitary nephrocyte. (a–a″) Podocytes in eucoelomates. Podocytes (P) form a filtration epithelium, through which primary urine is produced (a) and excluded via a nephridium. Foot processes are formed by cytoplasmic protrusion from the cell's periphery and are interdigitated with those of adjacent podocytes (a′). Thus, podocyte slit diaphragms between foot processes are regarded as an intercellular junction (aʹʹ). (b–b″) Nephrocytes in fruit fly (*Drosophila melanogaster*). Individual nephrocytes (N) are completely enwrapped by the basement membrane (brown). (b). Foot processes are formed by infolding of the basal plasma membrane (b′). Slit diaphragms are bridged between foot processes from the same nephrocyte, i.e., autocellular junction (b″). BM, basement membrane; FP, foot process; SD, slit diaphragm. Individual nephrocytes and podocytes are shown by different colors (purple and green)

Podocytes exhibit an efficient structure dedicated to the filtration of body fluid (Ichimura et al. 2017, 2007, 2019, 2015; Kriz and Kaissling 2007; Pavenstadt et al. 2003). In vertebrates, the large cell body of a podocyte projects several thick primary processes (Supplementary Fig. S2; Movie S1). The ridge-like prominences (RLPs) protrude from the cell body and primary processes to adhere themselves to the basement membrane. Furthermore, numerous fine foot processes protrude via RLPs. Adjoining podocytes are interdigitated by their foot processes, which keep regular intervals, filtration slits, between them. Moreover, adjacent foot processes are bridged with a unique intercellular junction, slit diaphragm, which functions as a selective barrier of the filtration (Fig. 1a–a″) (Assady et al. 2017; Ichimura et al. 2013, 2012).

Some groups of eucoelomates (Arthropoda, Onycophora, and Mollusca) possess nephrocytes, which are similar to podocytes in structure, in addition to podocytes (Crossley 1984; Haszprunar 1996; Seifert and Rosenberg 1977). Nephrocytes and podocytes commonly exhibit numerous fine foot processes bridged by slit diaphragms. The molecular components of these slit diaphragms are highly conserved between the nephrocytes in *Drosophila melanogaster* and podocytes in vertebrates (Weavers et al. 2009; Zhuang et al. 2009). Researchers in the field of podocyte biology and nephrology have recently taken advantage of these similarities to use *Drosophila* nephrocytes as a novel model system to investigate the function and disease of podocytes (Fu et al. 2017; Helmstädter and Simons 2017; Na et al. 2015; Tutor et al. 2014; Weide et al. 2017).

Although nephrocytes and podocytes are similar in several aspects, functionality and cellular architecture greatly differ between them (Fig. 1). Unlike podocytes, individual *Drosophila* nephrocytes are completely surrounded by basement membrane. Thus, they do not form an epithelial sheet and do not link to the Malpighian tubule, a modulating tubule peculiar to insets (Fig. 1b; Supplementary Fig. S1). Therefore, nephrocytes are not involved in the production of primary urine, but they serve to separate toxic molecules, such as heavy metals, from the hemolymph by endocytosis (Crossley 1984). Moreover, the formation of foot processes differs significantly between *Drosophila* nephrocytes and eucoelomate podocytes (Kawasaki et al. 2019). As mentioned above, the podocyte foot processes are formed by protrusion and are interdigitated between adjacent podocytes, and thus, the podocyte slit diaphragm is an intercellular junction (Fig. 1a–a″). In contrast, the nephrocyte foot processes are formed by infolding/invagination of the basal plasma membrane in *Drosophila*. Thus, as nephrocytes exist as solitary cells, the nephrocyte slit diaphragm is an autocellular junction in this animal (Fig. 1b–b″).

This enormous gap between solitary nephrocytes in *Drosophila* and epithelium-forming podocytes could be called a “missing link” in the evolution of nephrocytes and podocytes. In this study, to understand the evolution of nephrocytes, we have reevaluated the 3D structural diversity of nephrocytes in various taxonomic groups using focused-ion beam-scanning electron microscopy (FIB-SEM) tomography, a kind of volume scanning electron microscopy (Heymann et al. 2006; Kubota 2015; Ohno et al. 2015; Titze and Genoud 2016). We found that decapod nephrocytes exhibited structural similarity to podocytes higher than that of *Drosophila* nephrocytes, filling this missing link, and discussed the nephrocytes as part of the spectrum of filtration epithelial diversity in animal phylogeny.

## Materials and methods

### Animals

#### Decapod crustaceans

The decapod species examined are listed in Supplementary Table S1. Three species of marine decapods, i.e., prawn (*Marsupenaeus japonicus*), lobster (*Panulirus japonicus*), and hermit crab (*Aniculus miyakei*), collected in the Pacific coast of Japan were purchased from a local fish store. Two species of fresh-water decapods, crayfish (*Procambarus clarkii*) and mitten crab (*Eriocheir japonica*), collected in West Japan were purchased from a local pet shop. Decapod gills were isolated under anesthesia using a eugenol-based anesthetic agent FA100 (DS Pharma Animal Health, Osaka, Japan). The isolated gills of marine and fresh-water decapods were fixed in 2.5% glutaraldehyde solution buffered with 0.1 M and 0.2 M phosphate buffer (PB), respectively. Then, fixed samples were further immersed in the same fixative solution and stored at 4 °C.

#### Drosophila melanogaster

Adult flies of Canton-S strain were used to analyze the normal ultrastructure of the pericardial nephrocytes. Cultures were performed using standard fly food, and the flies were raised at 25 °C. Flies were hydrophilized using 0.1% Triton X-100/0.1 M PB after anesthesia with CO_2_ gas. Flies were dissected to isolate the heart and nephrocytes with dorsal body wall in 2.5% glutaraldehyde solution buffered with 0.1 M PB. The samples were further immersed in the same fixative solution and stored at 4 °C.

#### Rats

The 3D ultrastructure of podocytes from adult (10-week-old, male) Wistar rats (Charles River Japan, Yokohama, Japan) was compared with that of the branchial nephrocytes. The rats were perfused (under pentobarbital anesthesia) with physiological saline and then with 2.5% glutaraldehyde solution buffered with 0.1 M PB. The fixed specimens were further immersed in the same fixative solution and stored at 4 °C. These procedures were approved by the Institutional Animal Care and Use Committee of Juntendo University (approval no. 300226) and were carried out in accordance with the Guidelines for Animal Experimentation of Juntendo University. For the detailed protocol of perfusion fixation, see Ichimura et al. (2007).

### Combinatorial heavy metal staining for FIB-SEM tomography

Fixed specimens were processed using a combinatorial heavy metal staining protocol for enhancing the signal for the backscatter electron imaging of epoxy-resin-embedded biological samples at low accelerating voltages. In brief, the specimens were successively immersed in 1% osmium tetroxide which contained 1.5% potassium ferrocyanide in 0.1 M cacodylate buffer for 1 h on ice, 1% low molecular weight tannic acid (Electron Microscopy Sciences, Hatfield, PA) in 0.1 M cacodylate buffer for 4 h at 25 °C, 2% aqueous osmium tetroxide for 30 min at 25 °C, and 1% aqueous uranyl acetate overnight at 25 °C. Subsequently, samples were dehydrated with a graded series of ethanol and embedded in epoxy resin, Oken Epok 812 (Okenshoji, Tokyo, Japan). For the detailed protocol of sample preparation, see Miyaki et al. (2020a, b).

### Acquisition of serial block-face images using FIB-SEM tomography

Serial FIB-SEM images were obtained at 30 nm increments with a backscattered electron detector at 2.0-kV acceleration voltage using a Helios Nanolab 660 FIB-SEM (Thermo Fisher Scientific, Waltham, MA, USA). The pixel size of each FIB-SEM image was 13.5 × 17.1 nm (width × height × depth), and each recorded image was 3072 × 2048 pixels. Thus, the dimension of serial imaging by FIB-SEM was 41.5 × 35.0 μm (width × height). The new surface for serial FIB-SEM imaging was generated by FIB-milling using a 0.77-nA beam current, where gallium ions were accelerated with a voltage of 30 kV. For the detailed protocol of FIB-SEM tomography, see Kizilyaprak et al. (2014).

### Data processing for 3D reconstruction

Three-dimensional reconstruction of nephrocytes was performed using AMIRA 6.1 Software (Thermo Fisher Scientific) on a Mouse professional workstation (Mouse Computer Co. Ltd., Tokyo, Japan). The segmentation procedure was performed on a Cintiq 27QHD interactive pen display (Wacom, Tokyo, Japan).

## Results

Living decapod crustaceans (Decapoda) are classified into two suborders, Dendrobranchiata and Pleocyemata. The latter suborder is further divided into nine infraorders (Supplementary Fig. S3) (Tsang et al. 2008). In this study, we evaluated the 3D architecture of nephrocytes in five species of decapod crustaceans from five different taxonomic groups (Supplementary Fig. S3, Table S1) using FIB-SEM tomography. This technique enabled the efficient acquisition of a series of sectional images directly from resin-embedded decapod gill samples. The 3D architectures of nephrocytes could be evaluated from the reconstruction images obtained from a series of sectional FIB-SEM images.

### Sectional FIB-SEM images of nephrocytes in decapods

In the species examined, nephrocytes commonly existed in the lumen of the branchial efferent vessels, which transport the oxygenated hemolymph to the pericardial sinus surrounding the heart. (Supplementary Fig. S4).

The contrast-inverted FIB-SEM images achieved a quality comparable to conventional transmission electron microscopy images (Supplementary Fig. S5). Like in *D. melanogaster*, nephrocytes exhibited numerous fine foot processes and slit diaphragms bridging between them in the five decapod species. Nephrocytes also possessed numerous endosomes and lysosomes, which corresponded to its function isolating toxic materials from the hemolymph.

### 3D architecture of nephrocytes in decapods

Here, we thus first overviewed their 3D architecture (Figs. 2 and 3) and then described the findings in detail (Figs. 4, 5, 6, 7, 8, 9 and 10**; **Supplementary Movies S2–S11).

**Fig. 2:**
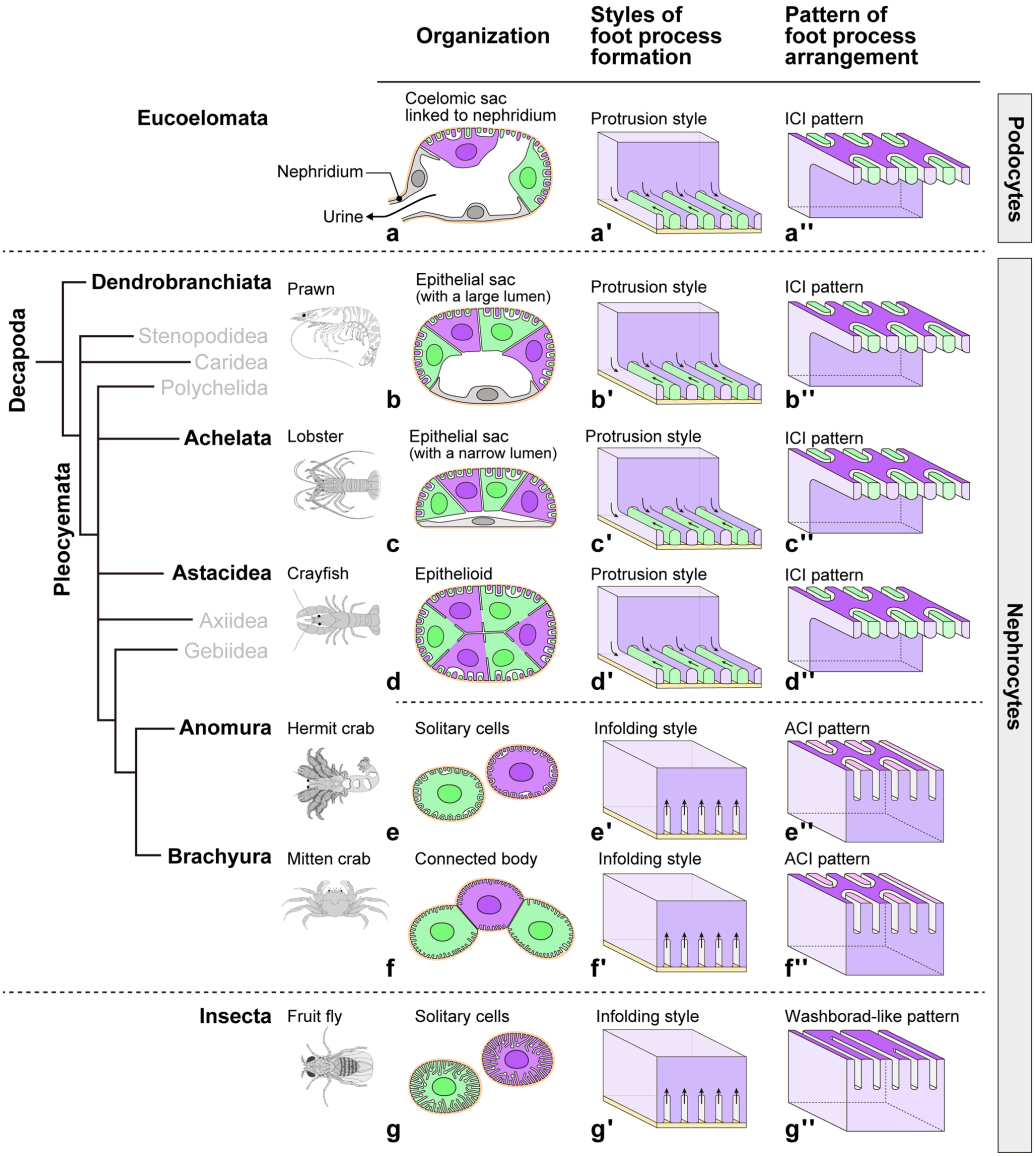
Architecture of decapod nephrocytes in relation to phylogeny. Decapod branchial nephrocytes exhibit higher similarity to the eucoelomate epithelium-forming podocytes (a–a″) than *Drosophila* solitary nephrocytes (g–g″) in tissue organization (a–g), foot process formation style (a′–g′), and pattern of foot process arrangement (a″–g″). (b–b″, c–c″, d–d″) In prawn, lobster, and crayfish, nephrocytes are organized into a closed epithelial sac or an epithelioid and foot processes are formed by protrusion and are arranged in an intercellular interdigitating (ICI) pattern as podocytes (a′, a″). (e–e″, f–f″) In hermit and mitten crabs, nephrocytes are not organized into an epithelium and foot processes are formed by infolding as in *Drosophila* nephrocytes (g′). However, foot processes are arranged following an interdigitating pattern, i.e., autocellular interdigitating pattern (ACI)

**Fig. 3:**
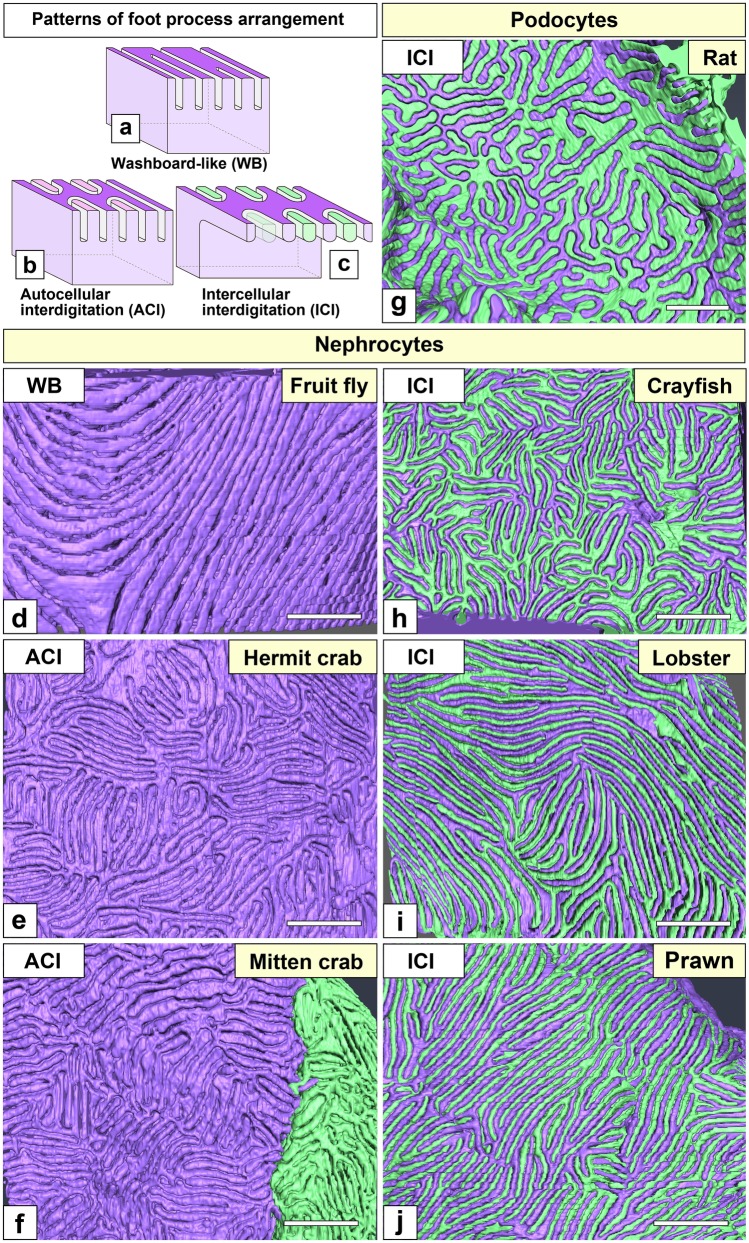
Patterns of foot process arrangement in nephrocytes. (a–c) Three patterns of foot process arrangement are recognized in nephrocytes. The washboard-like (WB) pattern is found in Fruit fly (*Drosophila melanogaster*) (a). In decapods, foot processes are arranged in an interdigitating pattern (b, c). In hermit and mitten crabs, foot processes from the same cell are interdigitated among them, i.e., autocellular interdigitating (ACI) pattern (b). In crayfish, lobster, and prawn, foot processes are interdigitated with those of adjacent cells, i.e., intercellular interdigitating (ICI) pattern (c). (d–f, h–j) Basal surface of 3D reconstructed nephrocytes in decapods. Individual nephrocytes are painted in different colors (purple and green). Foot process arrangement is clearly visible on the basal surface of 3D reconstructed nephrocytes. (g) Basal surface of 3D reconstructed podocytes in rat. Foot processes are arranged in an ICI pattern. Scale bars, 500 nm

**Fig. 4:**
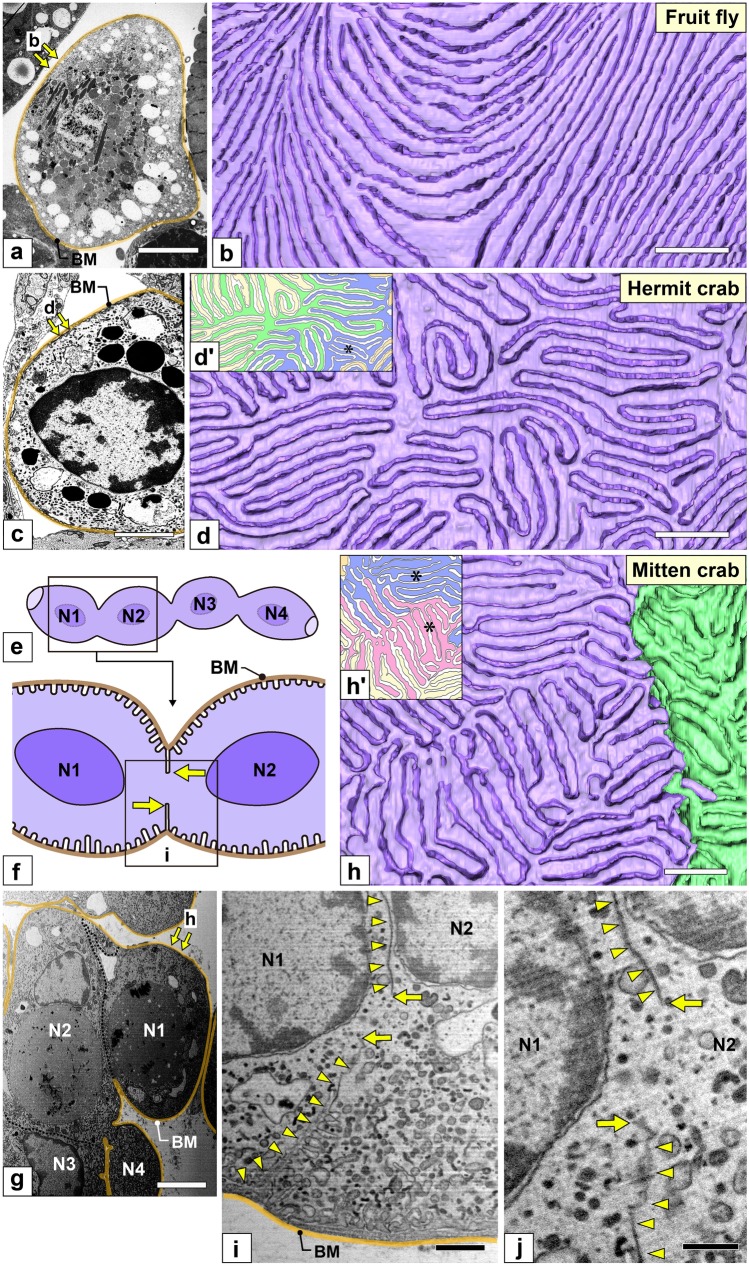
Nephrocytes in fruit fly, hermit crab, and mitten crab. (a, b) Fruit fly (*Drosophila melanogaster*). (c, d, d′) Hermit crab. (a, c) FIB-SEM sectional images. Nephrocytes existed as solitary cells without forming intercellular connections with each other in fruit fly and hermit crab. (b, d) The basal surface of reconstructed nephrocytes. The basal surface of foot processes, which adhered to the basement membrane, is painted by whitish-purple. (b) In fruit fly, foot processes ran linearly and both ends were usually anastomosed to the neighboring foot processes, i.e., they were arranged in a washboard-like pattern. (d) In hermit crab, only foot processes of the same nephrocyte were interdigitated, i.e., autocellular interdigitating (ACI) pattern. (e–j, h′) Mitten crab. (e, f) Schematic drawings. Multiple nephrocytes forming a connected body. The contacting membrane of adjacent nephrocytes is partially lost to form a cytoplasmic continuity (arrows). (g, i, j) FIB-SEM sectional images. (g) The connected body is surrounded by a basement membrane *en bloc* (brown line). (i) Adjacent nephrocytes in close contact with each other via planar intercellular junction (arrowheads) and form a cytoplasmic continuity (arrows). (j) Magnification of the cytoplasmic continuity shown in i. (h) The basal surface of a reconstructed connected body showing two adjacent nephrocytes (purple and green). Foot processes from the same cell interdigitated each other, but not those of the adjacent cells, i.e., autocellular interdigitating (ACI) pattern. (d′, h′) In hermit and mitten crabs, the basal surface of 20 to 30 foot processes formed an island (green, blue, red, and yellow regions). Adjacent islands were generally interdigitated by foot processes, but foot processes of the same island laid side by side in some regions (asterisk). BM, basement membrane; N1–N4, nephrocyte. Scale bars, 5 μm in a, c, g; 1 μm in b, d, h, i; 500 nm in j. The reconstructed nephrocytes (b, d, h) are also shown in Supplementary Movies S2–S4

**Fig. 5:**
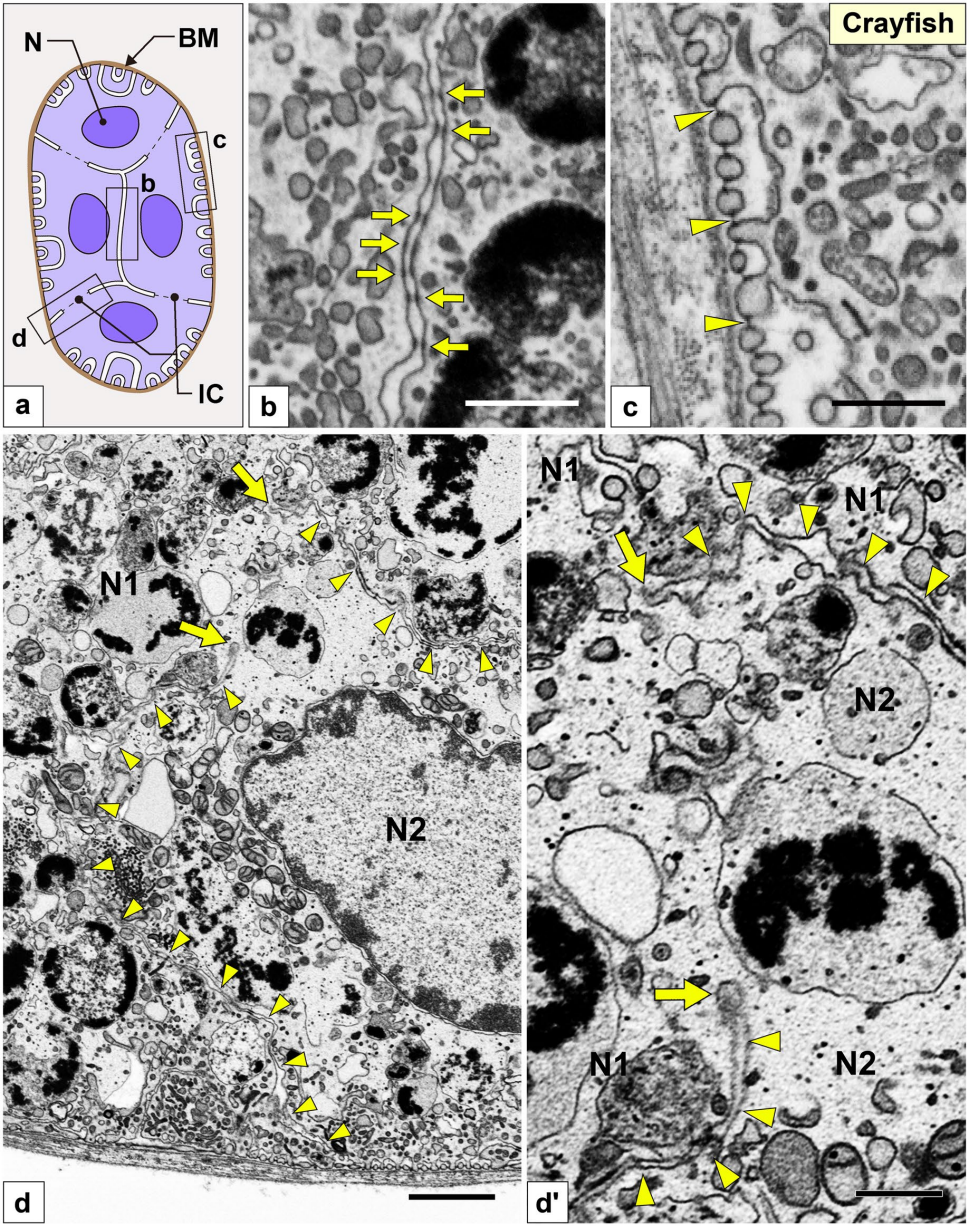
Nephrocytes in crayfish (I): FIB-SEM sectional images. (a) Schematic drawing of multiple nephrocytes forming an epithelioid, which is like an epithelial sac without lumen. The epithelioid is surrounded by a basement membrane *en bloc* (brown line). (b–d, d′) FIB-SEM sectional images. (b, c) The cell bodies of adjacent nephrocytes were closely apposed and connected via numerous spotty intercellular junctions (arrows in b), which were structurally different from the slit diaphragm (arrowheads in c). (d) The border of adjacent nephrocyte cell bodies (N1, N2) is indicated by arrowheads. The contacting membrane between adjacent cell bodies was partially lost to form a cytoplasmic continuity (arrows). (d′) Magnification of the cytoplasmic continuity shown in d. Scale bars: 1 μm in d; 100 nm in b, c, d′

**Fig. 6:**
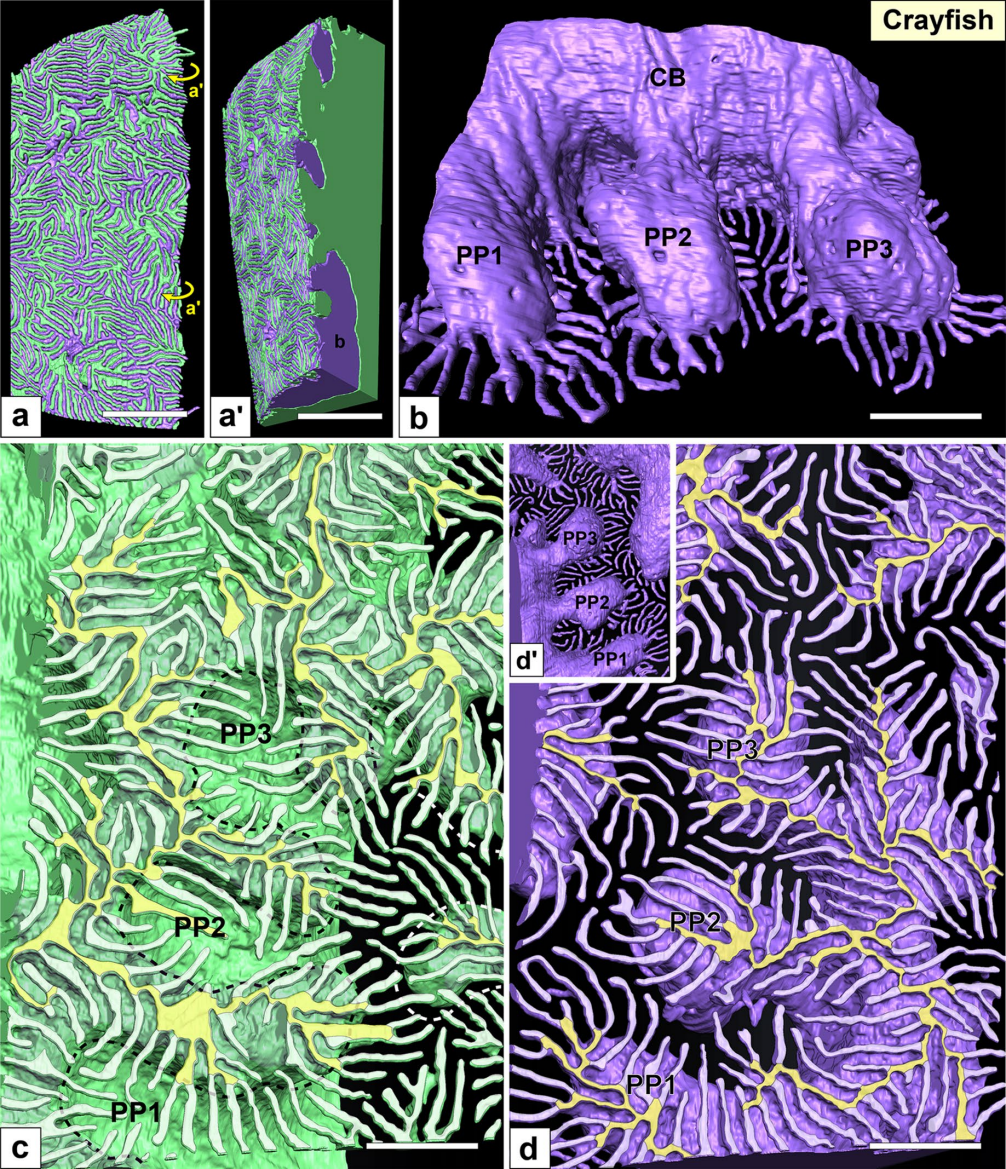
Nephrocytes in crayfish (II): reconstruction images. (a, a′) Reconstruction images of two adjacent nephrocytes (green and purple). Adjacent nephrocytes interdigitated each other. There was no obvious intercellular space between them. (b) Apical view of a single reconstructed nephrocyte. This cell is contained by the epithelioid shown in a, a′. Three primary processes (PP1–PP3), which typically exhibit a rounded shape, were projected from the periphery of the cell body (CB). (c, d) Basal view of reconstructed nephrocytes. Two adjacent reconstructed nephrocytes are separated. The purple cell shows the basal surface of primary processes (d). The green cell shows the basal surface of cell body, on which the primary processes of the adjacent purple cell were imprinted (dotted line in c). From the cell body and primary processes, numerous foot processes (whitish-green in c, whitish-purple in d) protruded via ridge-like prominences (yellowish-green in c, yellowish-purple in d). (d′) Apical view of the purple nephrocyte shown in d. Scale bars: 2 μm in a, a′; 500 nm in b–d. The reconstructed nephrocytes (b–d) are also shown in Supplementary Movies S5 and S6

**Fig. 7:**
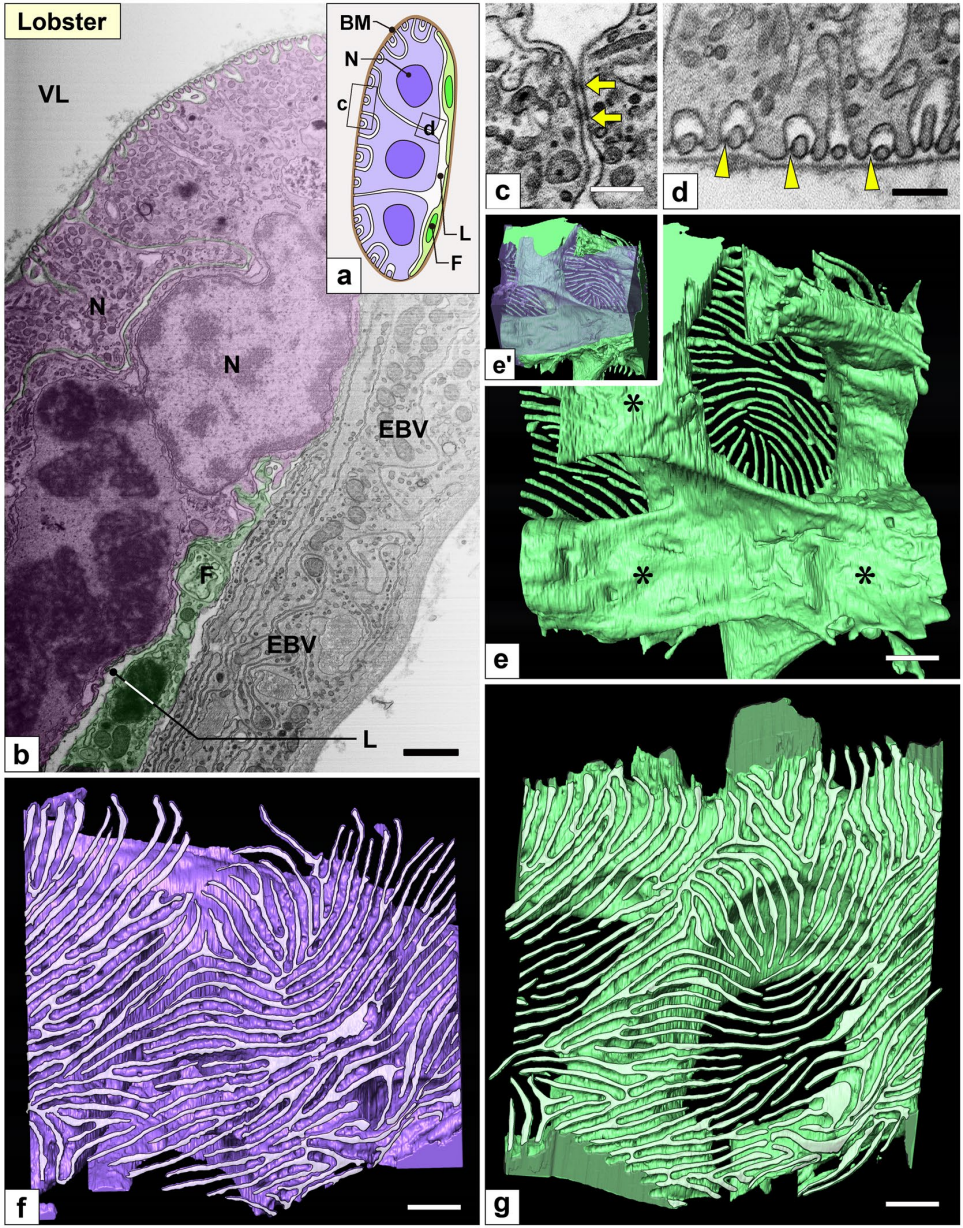
Nephrocytes in lobster (I): FIB-SEM sectional and reconstruction images. (a) Schematic drawing. Multiple nephrocytes (N) form an epithelial sac with a narrow lumen (L). The sac is partially altered into flat cells (F, green cells). There is no cytoplasmic continuity between nephrocytes. (b–d) FIB-SEM sectional images. (b) Connecting part of the nephrocytes (purple) and flat cells (green), which are in contact with the internal wall of the efferent branchial vessel (EBV). (c, d) The cell bodies and primary processes of adjacent nephrocytes were closely apposed and connected via spotty intercellular junctions (arrows in c), which were structurally different from the slit diaphragm between foot processes (arrowheads in d). (e–g, e′) Reconstruction images of two adjacent nephrocytes (green and purple). (e) Luminal view of the green nephrocyte showing its cell body was divided into three massive parts (asterisks). (e′) The green and purple cells interdigitated each other by these massive parts. (f, g) Basal view of purple and green nephrocytes. Numerous fine, long foot processes (whitish-purple in f, whitish-green in g) protruded from each massive part. Scale bars, 2 μm in b; 100 nm in c–g. The reconstructed nephrocytes (e–g) are also shown in Supplementary Movie S7

**Fig. 8:**
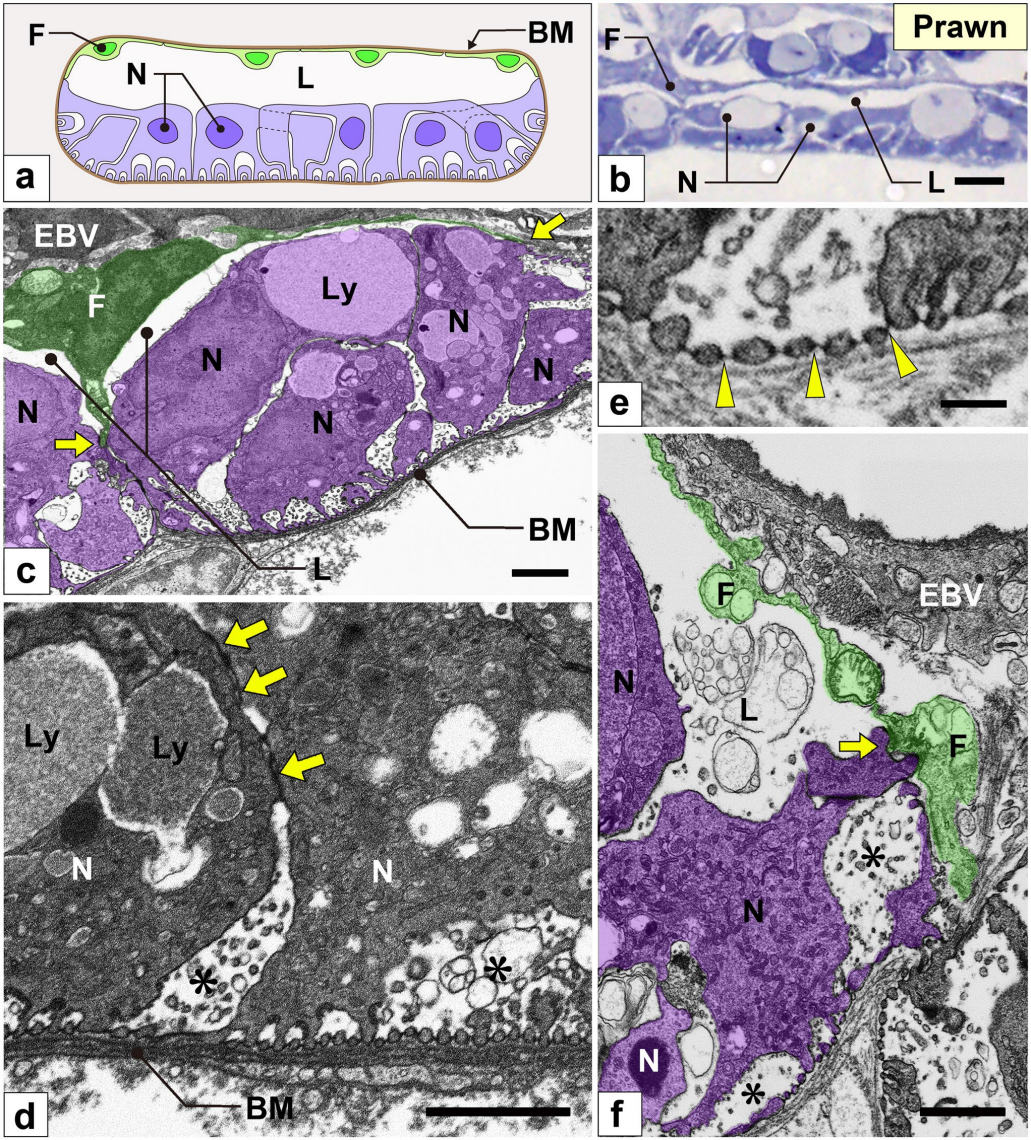
Nephrocytes in prawn (I): FIB-SEM sectional images. (a) Schematic drawing of multiple nephrocytes (N) forming a large epithelial sac with an obvious lumen (L). The sac is partially altered into flat cells (F, green cells). There is no cytoplasmic continuity between nephrocytes. (b) Semi-thin resin-section. The lumen of the epithelial sac is clearly visible with light microscopy. (c–f) FIB-SEM sectional images. (c, f) The connecting part (arrows) of nephrocytes (purple) and flat cells (green). Flat cells were in contact with the internal wall of the efferent branchial vessel (EBV). (d) Nephrocytes contained huge lysosomes (Ly). The cell bodies of adjacent nephrocytes were connected via spotty intercellular junctions (arrows in d) that are structurally different from the slit diaphragm between foot processes (arrowheads in e). Intercellular space (asterisks in d, f) was widely opened between nephrocytes. BM, basement membrane. Scale bars, 5 μm in b; 1 μm in c, d, f; 100 nm in e

**Fig. 9:**
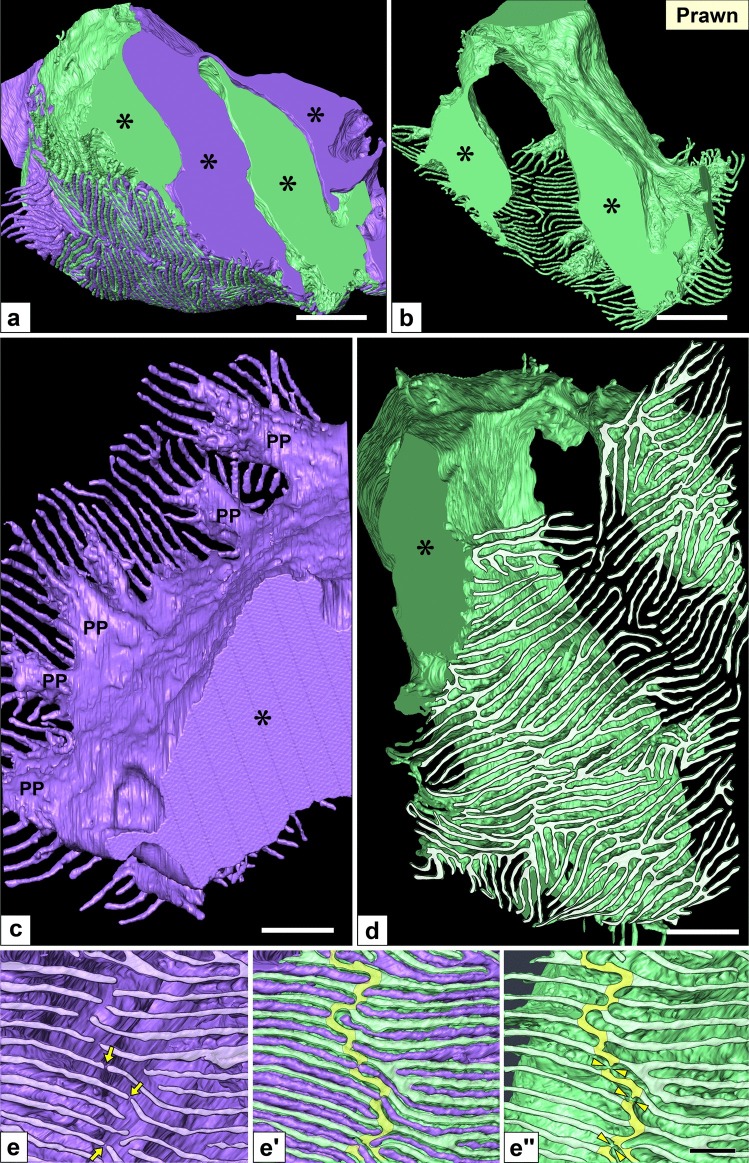
Nephrocytes in prawn (II): reconstruction images. (a–d) Reconstruction images of two adjacent nephrocytes (green and purple). (a, b) The cell body was divided into two massive parts (asterisks), while typically it is horseshoe-shaped (b). The massive parts were interdigitated between adjacent purple and green nephrocytes (a). (c) Luminal view of a massive part of the purple nephrocyte. Numerous fine long foot processes protruded from the massive part, some of which protruded via short thick primary processes (PP). (d) Basal view of the green nephrocyte, numerous fine long foot processes (whitish-green) protruded from each massive part. (e–e″) Magnification of the basal surface. Foot processes protruded via ridge-like prominences (RLP) (yellowish-green). RLP was partially retracted (arrowheads in e″). Thus, foot processes of the purple nephrocyte were closely apposed across the retracted RLP (arrows in e). Scale bars, 1 μm in a, b; 500 nm in c, d; 100 nm in e–e″. The reconstructed nephrocytes (a–d) are also shown in Supplementary Movies S8 and S11

**Fig. 10:**
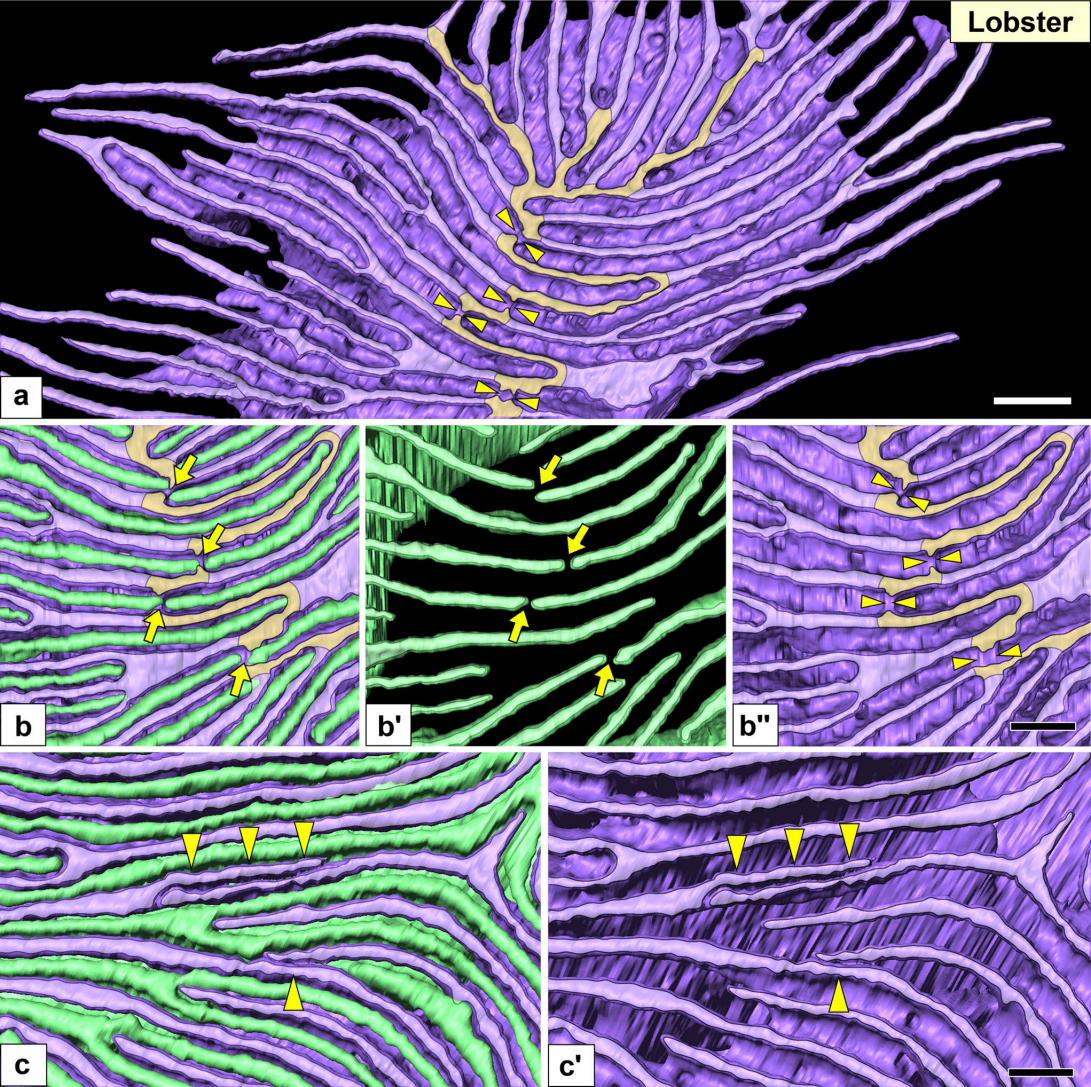
Nephrocytes in lobster (II): reconstruction images of foot processes and ridge-like prominences (RLPs). Reconstruction images of two adjacent nephrocytes (green and purple). All images show the basal surface of cells. (a) Numerous fine long foot processes (whitish-purple) protruded from a massive part via the RLP (yellowish-purple). (b–b″, c, c′) RLP was partially retracted (arrowheads in a, b″). Thus, foot processes were closely apposed across the retracted RLP (arrows in b, b′) or connected to form an autocellular junction (arrowheads in c, c′). Scale bars, 100 nm. The reconstructed nephrocyte (a) is also shown in Supplementary Movies S9 and S10

#### Overview

The nephrocytes were organized at different levels in each species, i.e., solitary/disjointed cells (like in *Drosophila* nephrocytes) (Fig. 2e, g), connected body (Fig. 2f), epithelioid (Fig. 2d), epithelial sac with narrow lumen (Fig. 2c), and epithelial sac with obvious lumen (Fig. 2b) in hermit crab, mitten crab, crayfish, lobster, and prawn, respectively. Moreover, the epithelial sac was quite similar to the podocyte-based coelomic sacs in invertebrate nephridial system (Fig. 2a).

Foot processes were formed in different ways in nephrocytes, i.e., infolding and protrusion styles. In the first one, foot processes formed by the infolding/invagination of the basal plasma membrane, and the slit diaphragm was formed between foot processes of the same cell as an autocellular junction (Fig. 2e′, f′), as found in *Drosophila* nephrocytes (Fig. 2g′). In the second one, foot processes formed by cytoplasmic extension, and the slit diaphragm was formed between foot processes of adjacent cells as an intercellular junction (Fig. 2b′, c′, d′), as found in eucoelomate podocytes (Fig. 2a′).

The arrangement of foot processes was largely different between *Drosophila* and decapods. *Drosophila* nephrocytes exhibited a washboard-like pattern, as previously reported (Fig. 3a, d) (Kawasaki et al. 2019), while decapod nephrocytes exhibited an interdigitating pattern, which could be intercellular (ICI) or autocellular (ACI) interdigitating pattern (Fig. 3b, c). The ICI pattern was found in crayfish, lobster, and prawn, in which nephrocytes were organized into the epithelioid or epithelial sac, and foot processes were interdigitated between adjacent nephrocytes (Fig. 3h–j) like in podocytes (Fig. 3g). The ACI pattern was found in hermit and mitten crabs, in which nephrocytes existed as solitary cells and connected body, respectively. The basal surface of 20–30 foot processes formed an “island,” and foot processes were interdigitated between the adjacent islands within the same nephrocyte (Fig. 3e, f).

Regarding the phylogenetic tree of decapod crustaceans, in Anomura and Brachyura (“crab”-called decapods), which are highly specialized groups in Decapoda, nephrocytes were not organized into an epithelium, and foot processes were formed by infolding and arranged in an ACI pattern (Fig. 2e–e″, f–f″). Meanwhile, in Dendrobranchiata, Achelata, and Astacidea (“shrimp”-shaped decapods), nephrocytes were organized into an epithelioid or epithelial sac, and foot processes were formed by protrusion and arranged in an ICI pattern (Fig. 2b–b″, c–c″, d–d″).

The structural features peculiar to each species were described in the following sections.

#### Hermit and mitten crabs (compared to *D. melanogaster*)

In hermit crab, nephrocytes were individually surrounded by the basement membrane like in *Drosophila* (Fig. 4a, c). Thus, they existed as solitary cells without forming intercellular connections. While, in mitten crab, multiple nephrocytes formed a connected body similar in shape to streptococcus bacteria (Fig. 4e–g). The connected body was surrounded by a basement membrane *en bloc*. Adjacent nephrocytes were in close contact with each other via planar intercellular junction without intercellular space (arrowheads in Fig. 4i). The contacting membrane between adjacent cells was partially lost to achieve cytoplasmic continuity, resulting in the connected body forming a syncytium (arrows in Fig. 4f, i, j).

In both hermit and mitten crabs, foot processes were formed by infolding like in *Drosophila* but arranged in an ACI pattern unlike in *Drosophila*, in which foot processes were arranged in a washboard-like pattern (Fig. 4b, d, h; Supplementary Movies S2–S4). The basal surface of 20−30 foot processes formed an island (colored regions in Fig. 4d′, h′). Adjacent islands were generally interdigitated by foot processes, but foot processes of the same island laid side by side in some regions (asterisks in Fig. 4d′, h′).

#### Crayfish

Multiple nephrocytes formed an epithelioid, which was like an epithelial sac without lumen (Fig. 5a). The epithelioid was individually surrounded by a basement membrane *en bloc*. The cell bodies of adjacent nephrocytes were closely apposed and connected via numerous spotty intercellular junctions (arrows in Fig. 5b) that were structurally different from the slit diaphragm between foot processes (arrowheads in Fig. 5c). Like in mitten crab, the contacting membrane between adjacent cell bodies was partially lost to form cytoplasmic continuity (arrows in Fig. 5d, d′), resulting in the epithelioid forming a syncytium.

The basic architecture was similar between crayfish nephrocytes and vertebrate podocytes. The cell bodies of nephrocytes projected primary processes, which typically exhibited a rounded shape (Fig. 6b, d′; Supplementary Movie S5) and went under the cell body of adjacent nephrocytes, resulting in primary processes forming their impressions on the cell body of the adjacent nephrocytes (dotted line in Fig. 6c).

Foot processes were formed by protrusion. Numerous fine foot processes protruded from both the cell bodies and primary processes via RLPs (yellowish-white in Fig. 6c, d) like in vertebrate podocytes. Foot processes were interdigitated between adjacent nephrocytes, resulting in foot processes arranged in an ICI pattern (Fig. 6a, a′; Supplementary Movie S6).

#### Lobster and prawn

In both species, multiple nephrocytes formed an epithelial sac with a lumen (Figs. 7a, b and 8a–c), which was in contact with the internal wall of the efferent branchial vessels (Figs. 7b and 8f). The region contacting the vessel wall was altered into flat cells (green cells in Figs. 7b and 8f), which were similar to the parietal epithelial cells of Bowman’s capsule in the vertebrate kidney. Especially, in prawn, the epithelial sacs of nephrocytes entirely lined the internal wall of the efferent branchial vessels, and their lumens were quite larger than those in lobster (Fig. 8b, f).

The cell bodies of adjacent nephrocytes were connected via numerous spotty intercellular junctions (arrows in Figs. 7c and 8d) that were structurally different from the slit diaphragm between foot processes (arrows in Figs. 7d and 8e). Unlike in crayfish, nephrocytes did not form a cytoplasmic continuity.

The cell body of nephrocytes was divided into two to four massive parts (Figs. 7e, g and 9a, b; Supplementary Movies S7, S8), one of which contained a nucleus. These massive parts were interdigitated between adjacent nephrocytes (Figs. 7e′ and 9a). Foot processes were formed by protrusion. From each massive part, numerous fine and long foot processes protruded via RLPs (Figs. 7f, 9c, and 10a; Supplementary Movies S9–S11) and interdigitated between adjacent nephrocytes, resulting in foot processes arranged in an ICI pattern.

The RLPs were partially retracted (arrowheads in Figs. 9e″ and 10b″), resulting in the two foot processes on both sides of the retracted RLP being closely apposed (arrows in Figs. 9e–e′ and 10b–b′) or connected to form an autocellular junction (arrowheads in Fig. 10c, c′). Such contact between the tips of foot processes are not found in normal vertebrate podocytes.

## Discussion

FIB-SEM tomography, including a reconstruction technique, allowed visualization of the structural diversity of decapod nephrocytes in two aspects tissue organization and cellular architecture. Decapod nephrocytes showed several steps of tissue organization from solitary cells to epithelial sac. The basic architecture of nephrocytes was highly likely determined by their level of tissue organization. In crayfish, lobster, and prawn, nephrocytes were organized into an epithelial sac and their foot processes were formed by protrusion, like podocytes (Supplementary Fig. S6). In mitten and hermit crabs, nephrocytes were not organized into an epithelium and their foot processes were formed by infolding as in *Drosophila*. However, foot processes were arranged in an interdigitating pattern like podocytes (Supplementary Fig. S6). These findings indicated that decapod nephrocytes exhibited higher structural similarity to podocytes than those of *Drosophila*, filling an enormous gap between the solitary nephrocytes in *Drosophila* and the epithelium-forming podocytes, which could be called a “missing link” in the evolutionary diversity of nephrocytes and podocytes. Furthermore, owing to this continuity between nephrocytes and podocytes, it becomes necessary to clarify the definition of nephrocytes. Here we propose the following definition: “Nephrocytes, a kind of highly specialized podocyte, link their foot processes by slit diaphragms like podocytes, but they are not involved in the production of primary urine because they lack direct connection to the modulating tubules.”

In Arthropoda, including Crustacea, a set of podocyte-based coelomic sac and nephridium plays a role in excretion as the nephridial system (Supplementary Fig. S1) (Ruppert and Smith 1988). The crustacean nephridial system is associated with segmental appendages, which have undergone various modifications in individual body segments, such as antennae, maxillipeds, pereiopods, and pleopods (Ax 2000; Ruppert et al. 2003). In decapod crustaceans, a pair of nephridial systems, called antennal/green gland, is associated with the second antennae, and their terminal ends open on the exoskeleton near the base of the antennae (Longshaw and Stebbing 2016). The gills are also derived from the segmental appendages (pereiopods) in decapod crustaceans, implying that the coelomic-sac primordial cells enter the gills via their vascular system and differentiate at the various levels of tissue organization within the gill. Some crustaceans in Cephalocarida, Syncarida, Copepoda, and Isopoda possess the rudimentary coelomic sac without excretory modulating tubules, which are associated with the segmental appendages (Hessler and Elofsson 1995; Hosfeld and Schminke 1997; Wägele and Walter 1990). From conventional electron microscopy-based analyses, the rudimentary coelomic sac is considered to be formed by podocytes, which are called “segmental extranephridial podocytes.” However, if our new definition of nephrocyte is adapted, these podocytes should be referred to as nephrocytes. The epithelial sac of nephrocytes found in crayfish, lobster, and prawn highly likely corresponds to this rudimentary coelomic sac.

Nephrocytes exist in limited eucoelomate phyla, i.e., Arthropoda, Onycophora, and Mollusca (Crossley 1984; Haszprunar 1996; Seifert and Rosenberg 1977), and the sectional ultrastructures of nephrocytes have been reported in a number of species that belong to these phyla (Boer and Sminia 1976; Crossley 1972; Goodman and Cavey 1990; Kokkinopoulou et al. 2014). In several insect species, nephrocytes have been reported to contain multiple nuclei (Crossley 1984); however, it is unclear whether such nephrocytes contain multiple nuclei per cell or if multiple nephrocytes form the connected body, epithelioid, or epithelial sac as found in decapod branchial nephrocytes. In Mollusca, solitary nephrocytes, also referred to as rhogocytes and pore cells, are disseminated broadly in the mantle and muscular tissues (Haszprunar 1996), but their 3D architecture has not been elucidated so far. However, several transmission electron microscopy images reported in previous researches indicate their architecture is likely to be similar to that of *Drosophila* nephrocytes (Boer and Sminia 1976; Kokkinopoulou et al. 2014). FIB-SEM tomography would be useful in revealing the precise 3D architecture of these nephrocytes.

Acoelomates and pseudocoelomates, which form no body cavity lined with mesothelium, produce primary urine by using the terminal cells of protonephridia as filtration epithelial cells like eucoelomates (Supplementary Fig. S1) (Wilson and Webster 1974). Terminal cells show large diversity in structure among taxonomic groups (Kieneke et al. 2008; Rohde 2001). For instance, in some planarians, the terminal cell is shaped like a test tube with numerous rectangular filtration fenestrations on its cytoplasmic wall (Ishii 1980; Nakamura et al. 2014), and in priapulids, the multiple terminal cells form an epithelial sheet with interdigitating podocytes (Kümmel 1964). It is difficult to elucidate the 3D ultrastructure of terminal cells by conventional electron microscopy because, as nephrocytes, the terminal cells are almost completely enwrapped by the basement membrane. FIB-SEM tomography would also be useful in revealing the precise 3D architecture of terminal cells and, subsequently, elucidating the structural diversity of filtration epithelial cells including terminal cells, podocytes, and nephrocytes.

## Conclusion

FIB-SEM tomography is a powerful tool for analyzing the 3D architecture of nephrocytes in more detail than that previously possible using conventional electron microscopy. Nephrocytes in decapod crustaceans filled the enormous gap in the evolutionary diversity of podocytes and nephrocytes. Thus, we conclude that the nephrocytes are part of the spectrum of structural diversity in filtration epithelia.

## Electronic supplementary material

Below is the link to the electronic supplementary material.

Supplementary file1 (DOCX 6668 kb)

Supplementary file2 (AVI 20471 kb)

Supplementary file3 (AVI 7337 kb)

Supplementary file4 (AVI 9388 kb)

Supplementary file5 (AVI 14265 kb)

Supplementary file6 (AVI 8738 kb)

Supplementary file7 (AVI 9431 kb)

Supplementary file8 (AVI 10520 kb)

Supplementary file9 (AVI 13531 kb)

Supplementary file10 (AVI 9918 kb)

Supplementary file11 (AVI 10789 kb)

Supplementary file12 (AVI 13946 kb)
