# Supplementary material for: Nephrocytes are part of the spectrum of filtration epithelial diversity
Source: Cell Tissue Res. 2020 Nov 16;382(3):609–25. doi: 10.1007/s00441-020-03313-7 (PMC7683493; doi:10.1007/s00441-020-03313-7)
Supplement: Supplementary file 1 — Supplementary file1 (DOCX 6668 kb) [file 441_2020_3313_MOESM1_ESM.docx]

**SUPPLEMENTARY INFORMATION**

**Nephrocytes are part of the spectrum of filtration epithelial diversity**

Takayuki Miyaki^1^, Yuto Kawasaki^1^, Akira Matsumoto^2^, Soichiro Kakuta^3^, Tatsuo Sakai^1^, and Koichiro Ichimura^1, 3*^

^1^ Department of Anatomy and Life Structure, Juntendo University Graduate School of Medicine, Tokyo, Japan;

^2^ Department of Biology, Juntendo University School of Medicine, Inzai, Chiba, Japan;

^3^ Laboratory of Morphology and Image Analysis, Center for Biomedical Research Resources, Juntendo University Graduate School of Medicine, Tokyo, Japan.

**Table of Contents:**

**Table S1.** Decapod crustaceans examined in this study.

**Figure S1.** Excretory system and nephrocytes in metazoan.

**Figure S2.** 3D architecture of vertebrate podocytes.

**Figure S3.** Phylogeny of decapod crustaceans.

**Figure S4.** Localization of nephrocytes and podocytes in decapod crustaceans.

**Figure S5.** FIB-SEM sectional images of nephrocytes.

**Figure S6.** Styles of foot process formation and arrangement of foot processes in nephrocytes.

**Movie S1.** 3D reconstructed podocyte from a normal adult rat.

**Movie S2.** 3D reconstructed pericardial nephrocyte from a fruit fly, *Drosophila melanogaster*.

**Movie S3.** 3D reconstructed hermit crab nephrocyte.

**Movie S4.** 3D reconstructed mitten crab nephrocytes.

**Movie S5.** 3D reconstructed crayfish nephrocyte (I).

**Movie S6.** 3D reconstructed crayfish nephrocyte (II).

**Movie S7.** 3D reconstructed lobster nephrocyte (I).

**Movie S8.** 3D reconstructed prawn nephrocyte (I).

**Movie S9.** 3D reconstructed lobster nephrocyte (II).

**Movie S10.** 3D reconstructed lobster nephrocyte (III).

**Movie S11.** 3D reconstructed prawn nephrocyte (II).

**Table S1.**

**Decapod crustaceans examined in this study.**

**

**

**
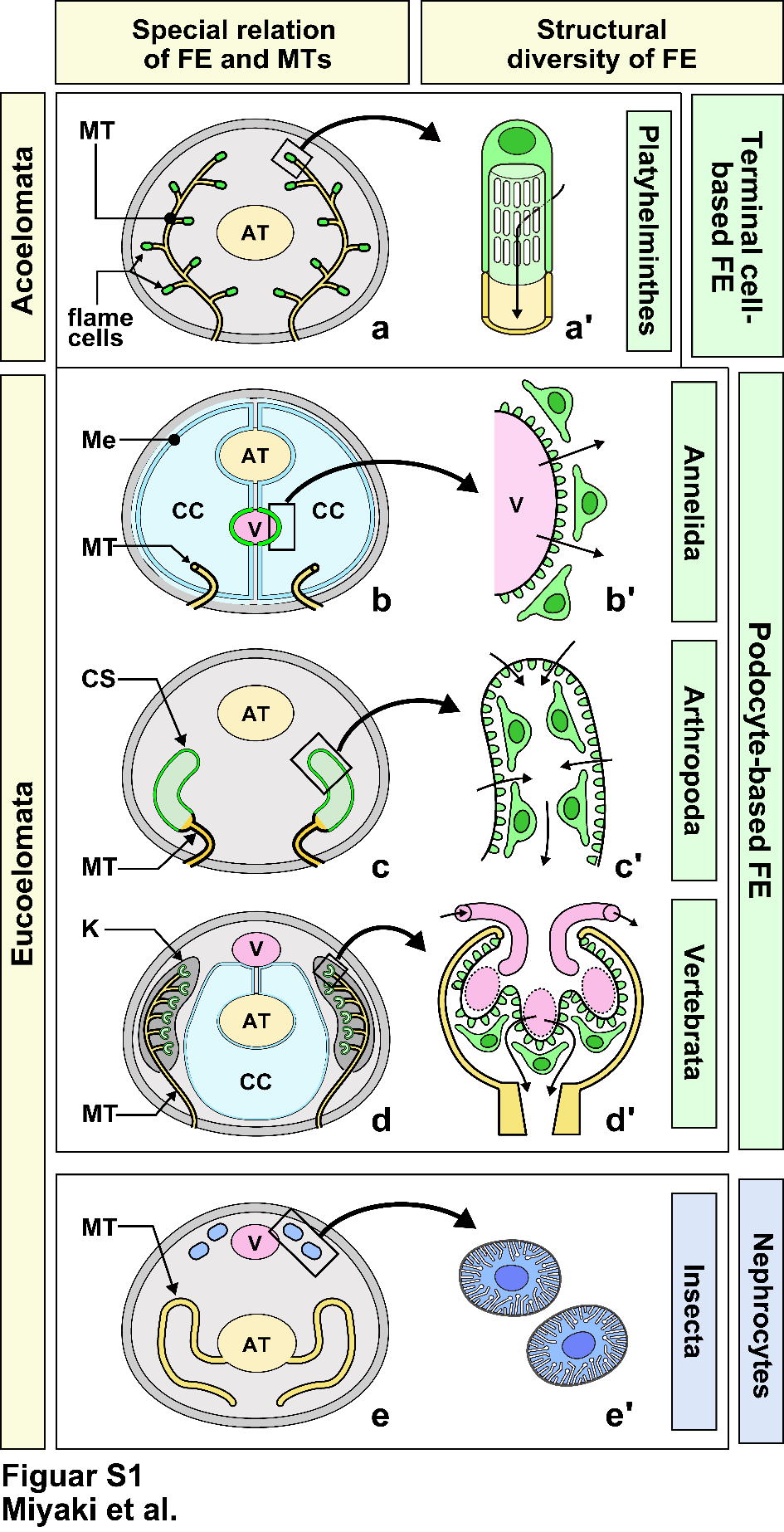
**

**Figure S1.**

**Excretory system and nephrocytes in metazoan.**

The excretory systems produce primary urine by ultrafiltration of body fluid via a filtration epithelium (**FE**) in general. Primary urine is subsequently transferred to a modulating tubule (**MT**), where the final urine is produced from primary urine by epithelial secretion and reabsorption. (**a, aʹ**) In acoelomata, FE is formed by terminal cells (colored in green), which links to an MT, i.e., protonephridium. (**b–d, bʹ–dʹ**) In eucoelomata, the mesothelium of the coelom (**b, bʹ**) or coelomic sac (**c, cʹ**) partially develops into FE formed by podocytes (in green). Primary urine excluded into the coelomic cavity enter into another kind of MT, i.e., nephridium. In vertebrates, the Bowman’s capsule, which contains the podocyte-based filtration epithelium, can be regarded as a micro-coelomic sac newly formed in the kidney (**d, dʹ**). (**e, eʹ**) Nephrocytes, a kind of podocyte-related cell, existed in limited eucoelomate phyla including Arthropoda. In *Drosophila*, nephrocytes (in blue) exist as solitary cells independent from the Malpighian tubule, an MT peculiar to insects. Thus, the nephrocytes are not involved in the production of urine. **AT**, alimentary tract; **CC**, coelomic cavity; **CS**, coelomic sac; **K**, kidney (mesonephros or metanephros); **V**, vasculature.

**
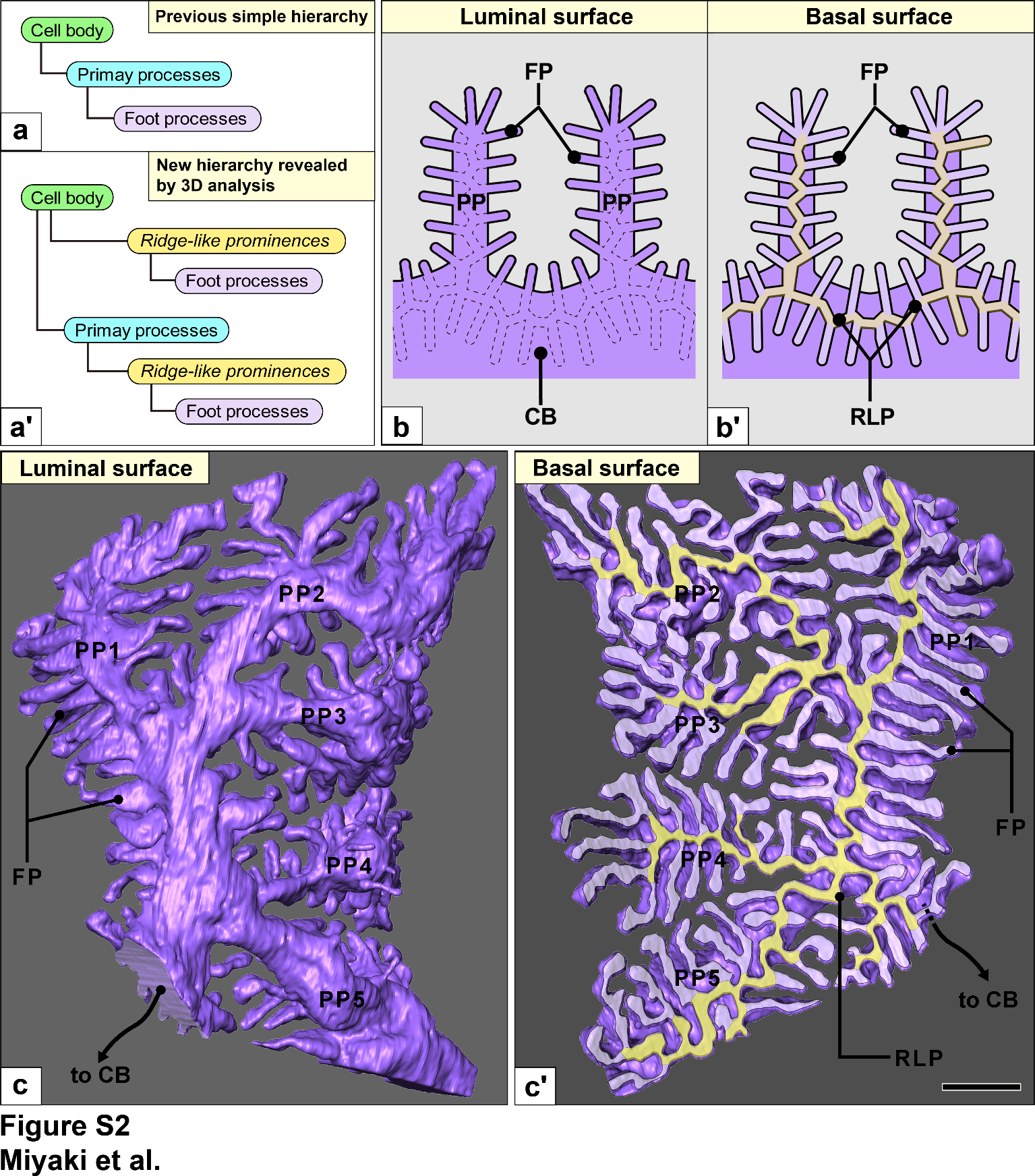
**

**Figure S2.**

**3D architecture of vertebrate podocytes.**

(**a, aʹ**) Structural hierarchy of podocyte subcellular compartments. In the classical textbooks, the three subcellular compartments of the podocyte, i.e., cell body, primary processes, and foot processes, are related in this order (**a**). From our 3D ultrastructural analysis using FIB-SEM tomography, it was revealed that podocytes contain a new subcellular compartment, the ridge-like prominence (RLP), which protrudes from the basal surface of the cell body and primary processes. Foot processes further protrude via the ridge-like prominences (RLPs) (**aʹ**). (**b, bʹ**) Schematic representation of podocyte subcellular compartments based on the new hierarchy. RLP is colored in yellow (**bʹ**). RLPs serve as an adhesion apparatus for the attachment of the cell body and primary processes to the basement membrane, and to connect foot processes to the cell body and primary processes. (**c, cʹ**) 3D reconstruction of a podocyte. The luminal (**c**) and basal (**cʹ**) surfaces of the reconstructed primary processes. RLP (yellow in **cʹ**) is clearly visible on the basal surface. **CB**, cell body; **FP**, foot process; **PP**, primary processes; **RLP**, ridge-like prominence. Scale bars: 2 μm in **cʹ**.

**
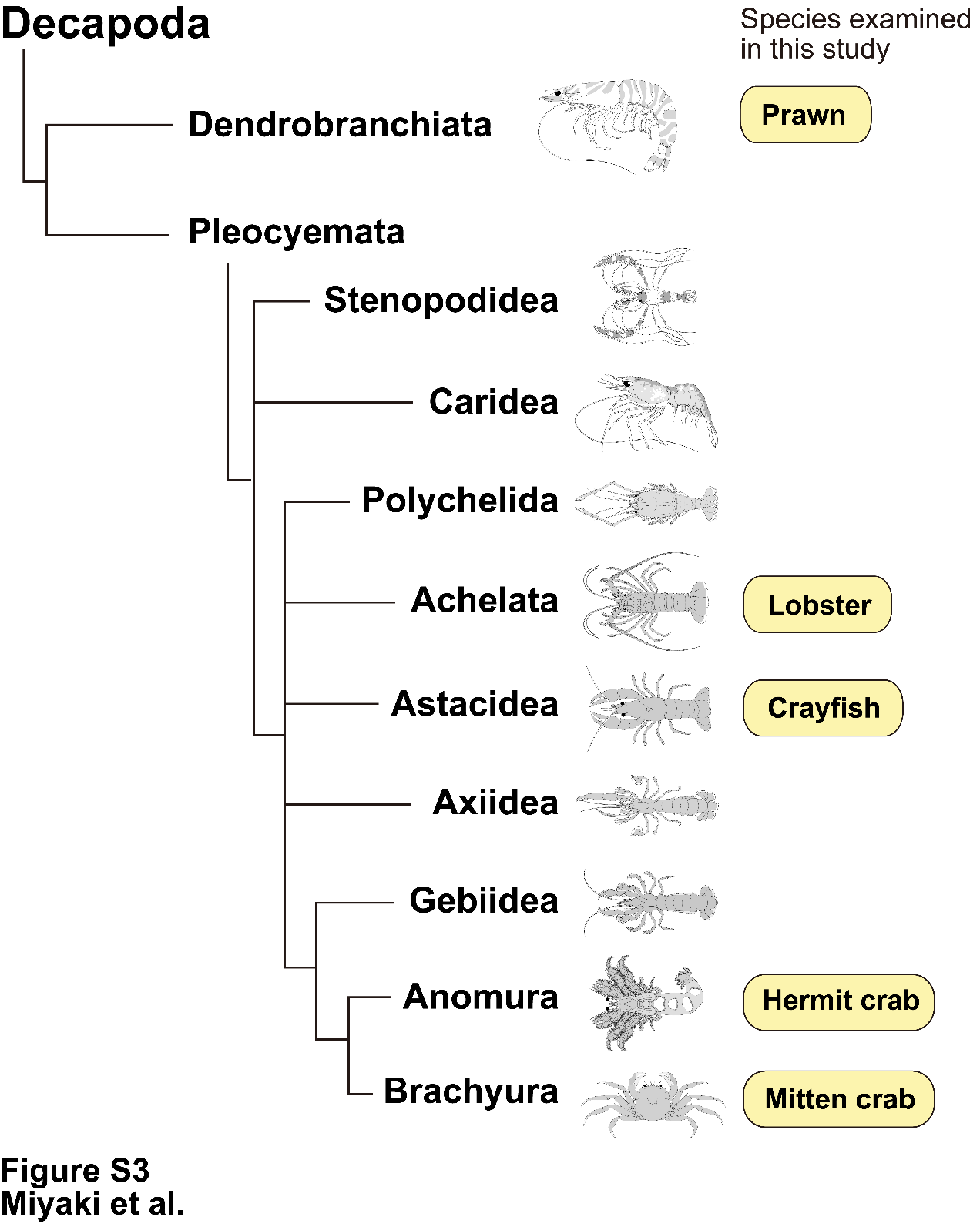
**

**Figure S3.**

**Phylogeny of decapod crustaceans.**

Decapod crustaceans (Decapoda) are classified into two suborders, Dendrobranchiata and Pleocyemata. Pleocyemata is further divided into nine infraorders. In this study, we examined the branchial nephrocytes in one Dendrobranchiata species (prawn) and four Pleocyemata species (lobster, crayfish, hermit crab, and mitten crab). The accurate names of the species examined are listed in **Table S1**. Dendrobranchiata is also divided into two infraorders, but these infraorders are not indicated in this tree.

**
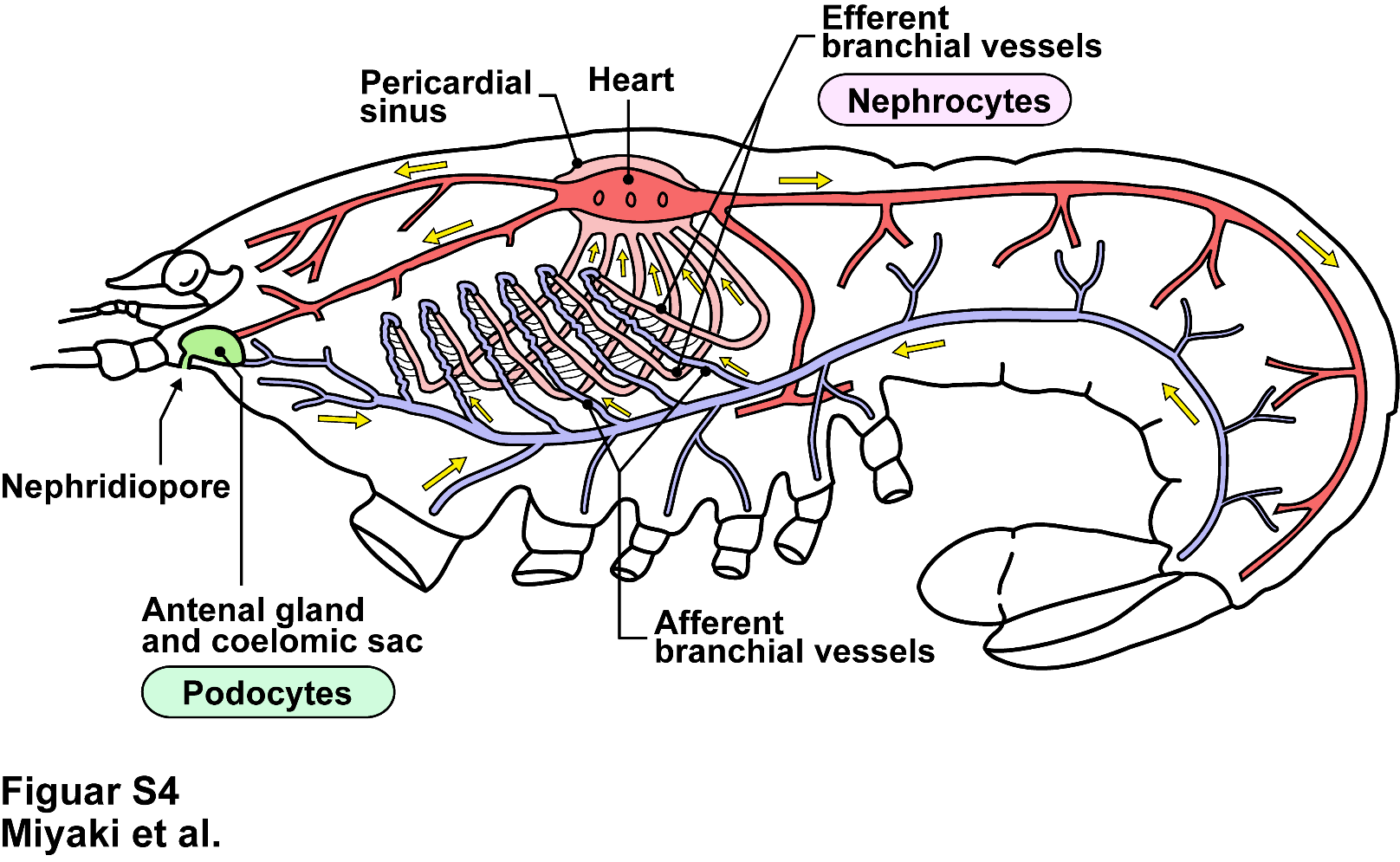
**

**Figure S4.**

**Localization of nephrocytes and podocytes in decapod crustaceans.**

Nephrocytes commonly existed in the lumen of the branchial efferent vessels, which transport the oxygenated hemolymph to the pericardial sinus surrounding the heart. Podocytes form the coelomic sac, where the primary urine is produced from blood by ultrafiltration. The primary urine is modified in the antennal gland and then carried out the body via nephridiopore. Arrows indicate the direction of blood stream.

**
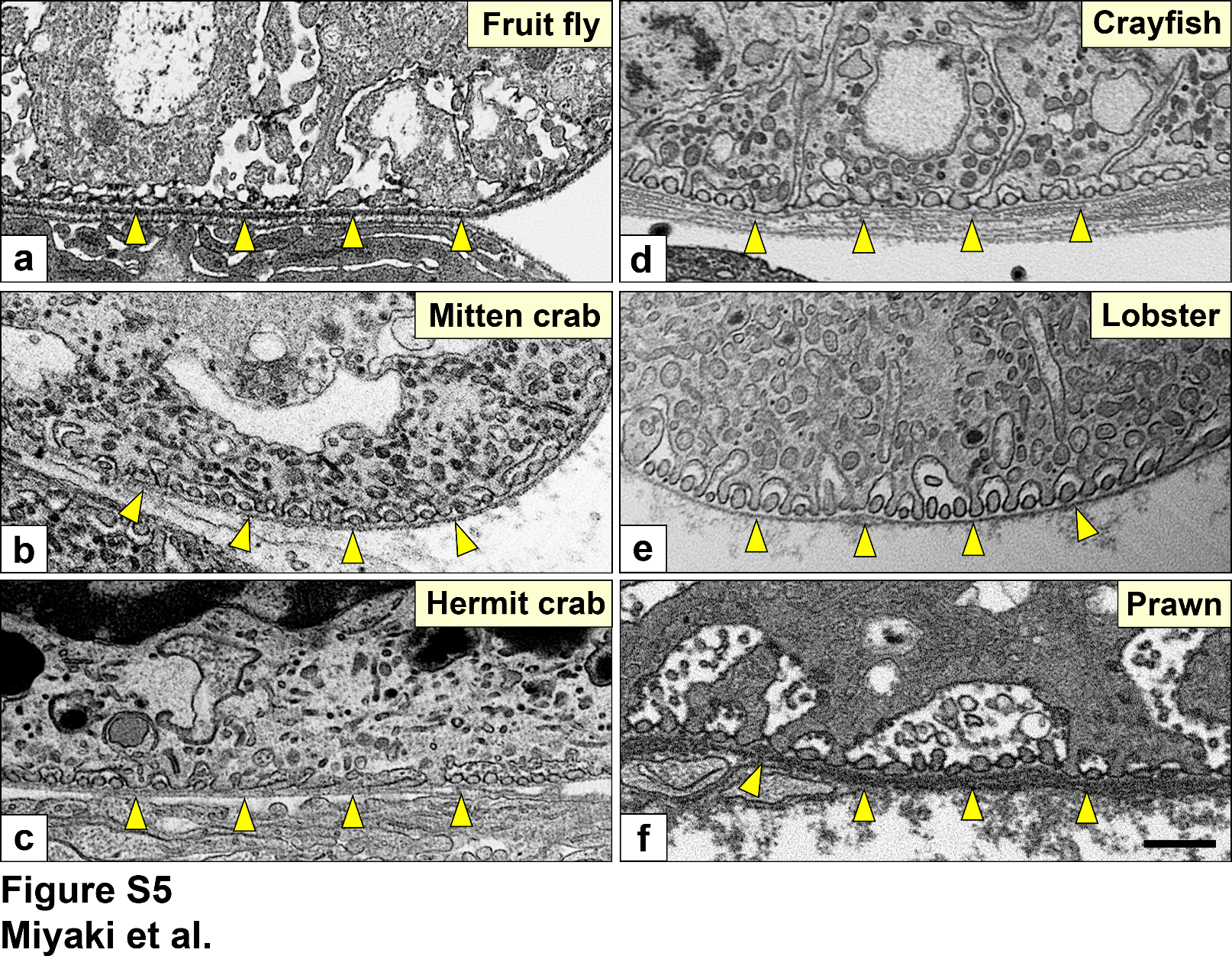
**

**Figure S5.**

**FIB-SEM sectional images of nephrocytes.**

Contrast-inverted FIB-SEM images of nephrocytes in fruit fly (*Drosophila melanogaster*) (**a**), mitten crab (**b**), hermit crab (**c**), crayfish (**d**), lobster (**e**), and prawn (**f**). These images are quite similar in quality to conventional transmission electron microscopy images. Foot processes and slit diaphragm are clearly visualized. Arrowheads, basement membrane of nephrocytes. Scale bars: 100 nm.

**
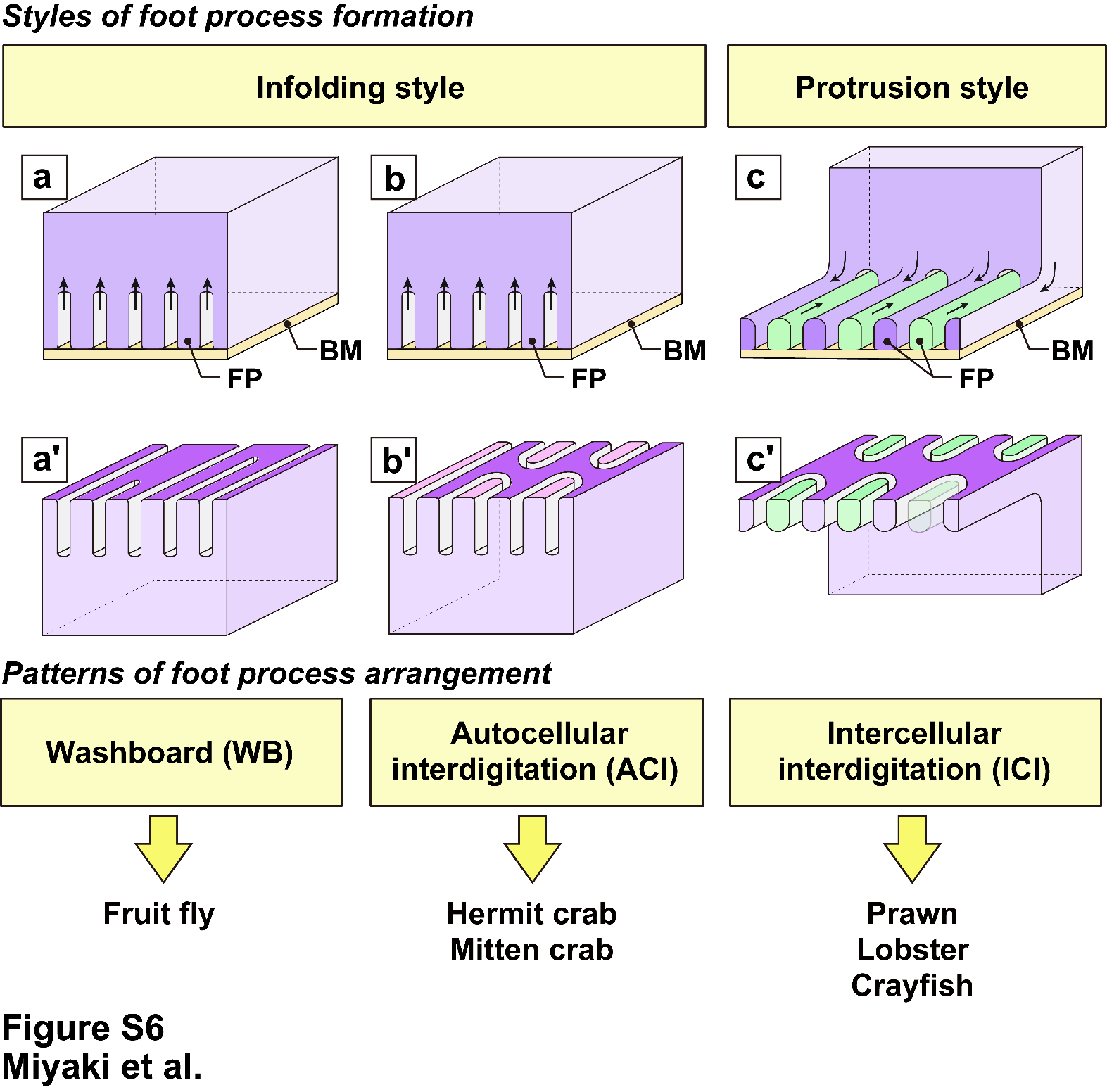
**

**Figure S6.**

**Styles of foot process formation and arrangement of foot processes in nephrocytes.**

(**a, aʹ**) In fruit fly (*Drosophila melanogaster*), the foot processes of nephrocytes are formed by the infolding style and arranged in a washboard-like pattern. (**b, bʹ**) In mitten and hermit crabs, foot processes are formed by the infolding style like in *Drosophila*. However, they are arranged in the autocellular interdigitating pattern. (**c, cʹ**) In crayfish, lobster, and prawn, foot processes are formed by the protrusion style and arranged in the intercellular interdigitation pattern like podocytes.

**Movie S1.**

**3D reconstructed podocyte from a normal adult rat.**

The reconstructed podocyte shown in **Fig. S2c, cʹ** is rotated around the vertical axis.

**Movie S2.**

**3D reconstructed nephrocyte from a fruit fly, *Drosophila melanogaster*.**

The reconstructed nephrocyte shown in **Figs. 3d, 4b** is rotated around the vertical axis.

**Movie S3.**

**3D reconstructed hermit crab nephrocyte.**

The reconstructed nephrocyte shown in **Figs. 3e, 4d** is rotated around the vertical axis.

**Movie S4.**

**3D reconstructed mitten crab nephrocytes.**

The reconstructed nephrocyte shown in **Figs. 3f, 4h** is rotated around the vertical axis.

**Movie S5.**

**3D reconstructed crayfish nephrocyte (I).**

The reconstructed nephrocyte shown in **Fig. 6b** is rotated around the vertical axis.

**Movie S6.**

**3D reconstructed crayfish nephrocyte (II).**

The reconstructed nephrocyte shown in **Fig. 6a, aʹ** is rotated around the vertical axis.

**Movie S7.**

**3D reconstructed lobster nephrocyte (I).**

The reconstructed nephrocyte shown in **Fig. 7e, eʹ, g** is rotated around the vertical axis.

**Movie S8.**

**3D reconstructed prawn nephrocyte (I).**

The reconstructed nephrocyte shown in **Fig. 9a, b** is rotated around the vertical axis.

**Movie S9.**

**3D reconstructed lobster nephrocyte (II).**

The reconstructed nephrocyte shown in **Fig. 7f** is rotated around the vertical axis.

**Movie S10.**

**3D reconstructed lobster nephrocyte (III).**

The reconstructed nephrocyte shown in **Fig. 10a** is rotated around the vertical axis.

**Movie S11.**

**3D reconstructed prawn nephrocyte (II).**

The reconstructed nephrocyte shown in **Fig. 9c** is rotated around the vertical axis.
